# The effect of muscle contusion on cortical bone and muscle perfusion following reamed, intramedullary nailing: a novel canine tibia fracture model

**DOI:** 10.1186/1749-799X-5-89

**Published:** 2010-11-30

**Authors:** Henry Koo, Thomas Hupel, Rad Zdero, Alexei Tov, Emil H Schemitsch

**Affiliations:** 1 Collingwood General and Marine Hospital, Collingwood, ON, Canada; 2 St. Mary's General Hospital and Grand River Hospital, Kitchener, ON, Canada; 3 Department of Mechanical and Industrial Engineering, Ryerson University, Toronto, ON, Canada; 4 Martin Orthopaedic Biomechanics Lab, St. Michael's Hospital, Toronto, ON, Canada; 5 Department of Surgery, Faculty of Medicine, University of Toronto, Toronto, ON, Canada

## Abstract

**Background:**

Management of tibial fractures associated with soft tissue injury remains controversial. Previous studies have assessed perfusion of the fractured tibia and surrounding soft tissues in the setting of a normal soft tissue envelope. The purpose of this study was to determine the effects of muscle contusion on blood flow to the tibial cortex and muscle during reamed, intramedullary nailing of a tibial fracture.

**Methods:**

Eleven adult canines were distributed into two groups, Contusion or No-Contusion. The left tibia of each canine underwent segmental osteotomy followed by limited reaming and locked intramedullary nailing. Six of the 11 canines had the anterior muscle compartment contused in a standardized fashion. Laser doppler flowmetry was used to measure cortical bone and muscle perfusion during the index procedure and at 11 weeks post-operatively.

**Results:**

Following a standardized contusion, muscle perfusion in the Contusion group was higher compared to the No-Contusion group at post-osteotomy and post-reaming (p < 0.05). Bone perfusion decreased to a larger extent in the Contusion group compared to the No-Contusion group following osteotomy (p < 0.05), and the difference in bone perfusion between the two groups remained significant throughout the entire procedure (p < 0.05). At 11 weeks, muscle perfusion was similar in both groups (p > 0.05). There was a sustained decrease in overall bone perfusion in the Contusion group at 11 weeks, compared to the No-Contusion group (p < 0.05).

**Conclusions:**

Injury to the soft tissue envelope may have some deleterious effects on intraosseous circulation. This could have some influence on the fixation method for tibia fractures linked with significant soft tissue injury.

## Background

Intramedullary nailing is the most widely used form of fixation for most open femoral and tibial shaft fractures [1-4]. Nailing allows for maintenance of bone length and alignment while reducing soft tissue disruption, relative ease of implant insertion, preservation of hematoma associated with fracture, and load sharing between the injured host bone and the inserted nail [5,6]. Healing rates for femur fractures treated with intramedullary nailing have been reported to be between 90 and 95% [5].

Reaming of the intramedullary canal to receive a nail in order to treat femoral and tibial shaft fractures associated with severe soft tissue injury remains controversial, since there are a number of negative consequences associated with reaming. Although intramedullary reaming allows the passage of a larger diameter nail, thereby providing more biomechanical stability because of better bone on nail contact [7-10], there are well-recognized deleterious effects of standard reaming on cortical bone blood perfusion [4,11-17]. However, it should be noted that cortical blood flow may be restored some weeks later. Reaming can also compromise the endosteal circulation when the surrounding soft tissue envelope becomes the principle source of blood supply for fracture healing [4,18-23]. Previous studies by some of the current authors have demonstrated the effects of reaming and canal fill on the blood flow to the tibia and its surrounding muscle [24,25]. Limited reaming, though, may be performed to minimize this effect [26]. However, these studies were done using a normal soft tissue envelope and are not representative of most clinical scenarios that might be encountered, highlighting the need for an investigation on reaming within an injured soft tissue envelope.

The purpose of this study, therefore, was to evaluate the effects of a standardized muscle contusion on blood flow to the tibia and its surrounding muscle following limited, intramedullary reaming and nail insertion of a segmental tibia fracture in a canine. In addition, by creating a reproducible standardized muscle contusion model, further studies could then be performed to evaluate other variables in this setting. It was hypothesized that bone and muscle blood perfusion would decrease due to tibial reaming and nailing to repair a segmental tibial fracture in the presence of a standardized muscle contusion.

## Methods

### Preoperative Period

Eleven adult mongrel dogs were used, each having a mass of least 24 kg. Each dog was conditioned into good health for a minimum of 21 days. Radiographs of both limbs were obtained preoperatively to ensure skeletal maturity and to measure canal diameter. This experimental protocol was approved by the animal care committee at the authors' institution. The initial study design was comprised of two groups of 6 animals each, however, one animal died prematurely and could not be included in the study.

### Experimental Groups

The 11 canines were distributed into two operative groups with no statistical difference in canal diameter between them. Although randomized allocation may be considered ideal, the present small sample size required non-randomized distribution to ensure equivalency between test groups. The Contusion group consisted of reamed intramedullary nailing (6.5 mm × 170 mm nail with reaming to 7.0 mm) with a standardized muscle contusion to the anterior muscle compartment (n = 6). The No-Contusion group consisted of reamed intramedullary nailing (6.5 mm × 170 mm nail with reaming to 7.0 mm) without muscle contusion (n = 5).

### Surgical Technique

Amoxicillin trihydrate/Clavulanate potassium (15 mg/kg PO BID) was given to the animals 48 hours prior to each surgical procedure. After sedation with subcutaneous Atropine sulfate (0.05 mg/kg) and Acepromazine maleate (0.03 mg/kg), anaesthesia was induced with intravenous Thiopental sodium (12.5 mg/kg), and Oxymorphone hydrochloride (0.05 mg/kg). After endotracheal intubation, general inhalational anesthesia was maintained with Halothane (1.5%), Nitrous Oxide (33%), and Oxygen (65.5%). Prophylactic Cefazolin (1 g IV) was administered at the beginning of the procedure and every two hours during the operation. Fluid requirements were maintained by intravenous Lactated Ringer's solution at 30 cc/kg/hr.

The left hindlimb of each animal was shaved, scrubbed, and prepped using 4% Chlorhexidine gluconate and 10% Povidone-iodine topical solution. No tourniquet or traction was used. The animals were placed in the supine position in a trough. A craniolateral approach to the tibia was made, extending from the lateral femoral condyle to the hock joint (ankle joint). The muscle fascia was incised. The muscle was then reflected, and the periosteum was elevated off the anterolateral aspect of the tibia. Blood flow to the muscle and bone were taken using a PF3 Laser Doppler Flowmeter (Perimed, Jarfalla, Sweden) with ± 10% accuracy and ± 3% precision in perfusion units. Measurements were taken of the cranial tibialis (tibialis anterior) muscle and the cortical bone at pre-specified locations, namely, 5.0, 8.5, and 15.0 cm distal to the lateral tibial plateau. The values were then averaged, as done in previous studies on canines by some of the current authors [24,25]. The measurements were taken by placing the laser doppler flowmetry probe on the surface of the muscle or bone until a stable reading was obtained. Perfusion measurements were then recorded for 60 seconds using a personal computer (Samsung, Notemaster, 486P; Samsung SDS America Inc., San Jose, CA, USA) and Perisoft software (Perimed, Jarfalla, Sweden).

**Figure 1:**
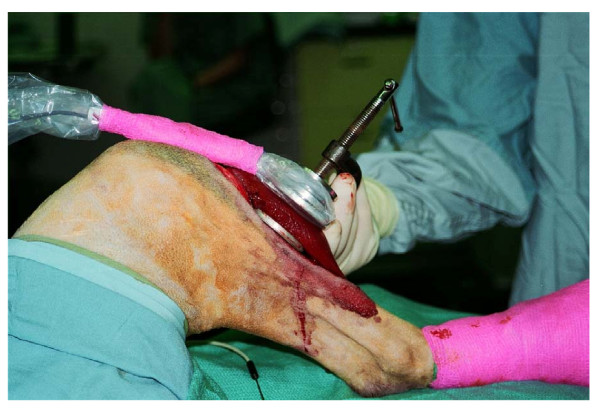
**Muscle contusion**. Intraoperative photograph of the anterior muscle compartment undergoing contusion prior to osteotomy using two circular aluminum discs, each with an area of 19 cm^2 ^mounted to the C-clamp. A 400 N force was applied for 20 sec. A strain gage meter was attached to the C-clamp to monitor the force reading.

Six dogs had the anterior muscle compartment contused using two aluminum discs, each with an area of 19 cm^2^, mounted on a C-clamp (Figure 1). A uniform force of 400 N was applied for 20 seconds. A calibrated strain gage meter (Infinity C Strain Gage Meter, Newport Electronics Inc., Santa Ana, CA, USA) quantified the force. The entire anterior compartment was contused at the level of the osteotomy. The contusion was performed prior to osteotomy. The application of contusion load using the C-clamp apparatus was performed identically for all tests. Thus, the relative effect of the C-clamp was the same for each test group.

Two osteotomies were performed to create a 2.5 cm mid-diaphyseal bone segment, as done previously by some of the authors in canine tibial fracture studies [24,25]. The proximal osteotomy site was 8.0 cm distal to the lateral tibial plateau. Complete subperiosteal dissection was carried out on the 2.5 cm segment to remove it from the surgical field. This devascularized segment was then re-introduced and reduced anatomically. The canal was sequentially reamed to 7.0 mm. The fracture was then stabilized with a custom-designed, solid 6.5 mm intramedullary nail made of 316L stainless steel (Synthes Canada, Mississauga, ON, Canada) locked proximally and distally with 2.7 mm screws.

In a standardized fashion from animal to animal, laser doppler flowmetry measurements were taken at ambient room temperature during the 2-hour surgical procedure, while the animal was anaesthetized, at four time intervals (pre-muscle contusion, post-osteotomy, post-reaming, and post-nailing) at the three sites previously specified. The values were then averaged, as done in prior studies on canines by some of the current authors [24,25]. The wound was closed in layers. The muscle fascia was left open to prevent the development of compartment syndrome. Subcutaneous tissue and skin were closed primarily. Sterile compression dressing was applied to the limb. During the perioperative period, the dogs were monitored at frequent intervals.

### Post-operative Period

Post-operative care included prophylactic Amoxicillin trihydrate/Clavulanate potassium (15 mg/kg PO BID for 7 days) and analgesia with Buprenorphine hydrochloride (0.02 mg/kg SC OD for 2 days). Wounds were monitored daily. The dogs were able to fully weight bear immediately. Standard anteroposterior and lateral radiographs of the left tibiae were taken at three-week intervals to assess fracture healing. Prior to radiography, sedation was provided using intravenous Oxymorphone hydrochloride (0.05 mg/kg).

### Week 11 Procedure

At 11 weeks post-operatively, general anaesthesia was induced according to the previously described protocol. A repeat craniolateral incision was made through the initial surgical incision. Final laser doppler flowmetry measurements at the sites previously specified were taken of the cortical bone and muscle. The animals were then euthanised with an overdose of intravenous Thiopental sodium (500 mg) and Potassium chloride. Bilateral tibiae were then harvested for radiographic analysis. This 11-week time point was chosen in order to be well beyond the point of bone union at the fracture site, which is known to begin at about 6 weeks post-operatively [27,28].

### Statistical Analysis

A similar approach to the statistical analysis described here was used in prior related studies on canines by some of the current authors [24,25]. Overall muscle and cortical blood flow were calculated as the average laser doppler flowmetry reading at the three measurement sites, namely, 5.0, 8.5, and 15.0 cm distal to the lateral tibial plateau. All perfusion values from laser doppler flowmetry were normalized by dividing average values by the baseline average value for both muscle and bone scenarios. Statistical comparisons were made between No-Contusion and Contusion groups for overall and segmental measurements at each time using paired t-tests with a p < 0.05 significance level. However, for subsequent comparisons between each time point with respect to baseline for each of the two muscle condition groups, non-paired t-tests were employed with an adjusted Bonferroni significance level of p < 0.01 in order to avoid type I error due to multiple comparisons. This adjusted value was calculated by dividing the p-value for a 95% confidence interval by the number of time points compared, i.e., p-value (Bonferroni) = p-value for 95% confidence interval/number of time points = 0.05/5 = 0.01.

### Post Hoc Power Analysis

A post hoc power analysis was performed to assess whether 5 specimens (No-Contusion) and 6 specimens (Contusion) per group were adequate to detect all statistical differences that might actually exist between these two contusion conditions, i.e. to avoid type II error, at a given time point. The computation for power using a one-tailed test was done at the 11-week time point for both muscle perfusion (overall) and bone perfusion (intercalary segment), since this most closely represents the long-term post-operative situation and is ultimately of interest to both clinicians and patients.

## Results

### Preoperative Data

There were no differences between the canal diameters of the two groups (p = 0.17). Average canal diameters were 8.8 ± 1.8 mm and 7.5 ± 1.0 mm for the No-Contusion and Contusion groups, respectively.

### Initial Intraoperative Data

**Figure 2:**
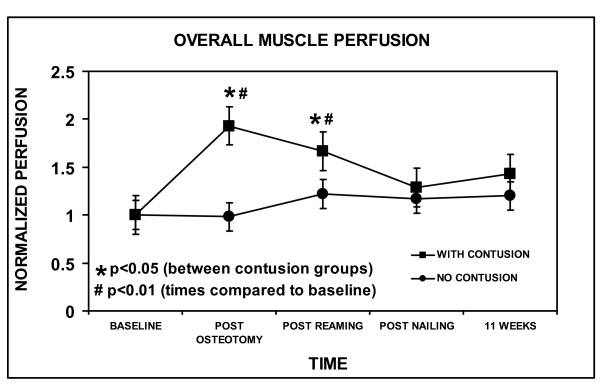
**Overall muscle perfusion**. Values for each time point were the average reading from the three different laser doppler flowmetry measurement locations. The values at each time point were all normalized by dividing by the average value at baseline. The error bars indicate one standard error of the mean. Statistically significant differences between Contusion and No-Contusion groups are indicated (*, p < 0.05). Statistical differences present when comparing procedures at each time to baseline only occurred in the Contusion group (#, p < 0.01).

Immediately following standardized contusion, overall muscle perfusion in the Contusion group was higher compared to the No-Contusion group at post-osteotomy and post-reaming (Figure 2). Overall muscle perfusion increased nearly two-fold in the Contusion group at post-osteotomy compared to baseline.

**Figure 3:**
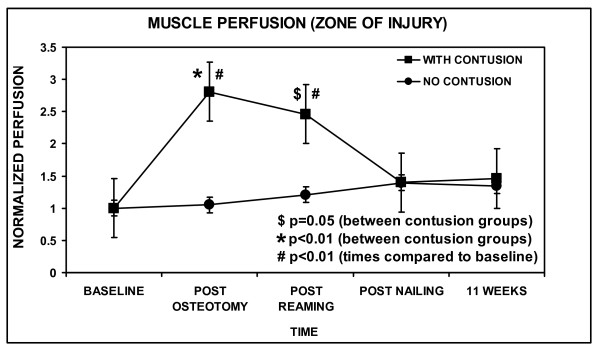
**Muscle perfusion within the zone of injury**. Values for each time point were the average reading from the three different laser doppler flowmetry measurement locations. The values at each time point were all normalized by dividing by the average value at baseline. The error bars indicate one standard error of the mean. Statistically significant differences between Contusion and No-Contusion groups are indicated (*, p < 0.01). A borderline statistical difference was found at post-reaming ($, p = 0.05). Statistical differences existed when comparing procedures at each time to baseline only in the Contusion group (#, p < 0.01).

Site-specific analysis revealed that most of this difference was found within the injury zone, where the muscle perfusion was found to be nearly three times higher than the baseline value in the Contusion group (Figure 3). In the Contusion group, there was a statistically significant decrease in the muscle perfusion following reaming with respect to baseline. There remained a borderline significant difference between the two groups following reaming (p = 0.05). Towards the end of the initial procedure (post-nailing), the increase in muscle perfusion in the Contusion group returned to nearly normal levels. In the No-Contusion group, muscle perfusion returned to baseline value. There was no longer any significant difference in muscle perfusion between the two groups at the end of the procedure (p = 0.45), which had both returned to baseline values.

**Figure 4:**
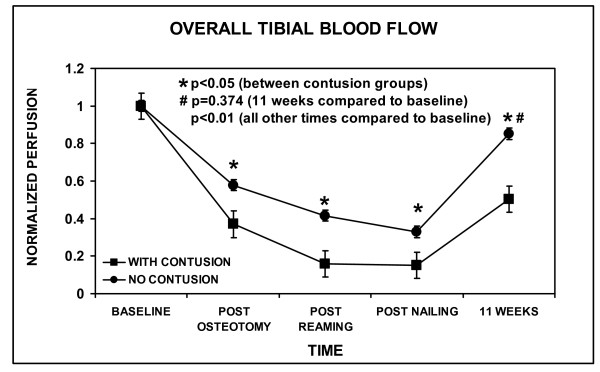
**Overall cortical bone blood flow of the tibia**. Values for each time point were the average reading from the three different laser doppler flowmetry measurement locations. The values at each time point were all normalized by dividing by the average value at baseline. The error bars indicate one standard error of the mean. Statistically significant differences between Contusion and No-Contusion groups are indicated (*, p < 0.05). All procedures at each time compared to baseline were statistically significant (p < 0.01), except for 11 weeks in the No-Contusion group (#, p = 0.374).

**Figure 5:**
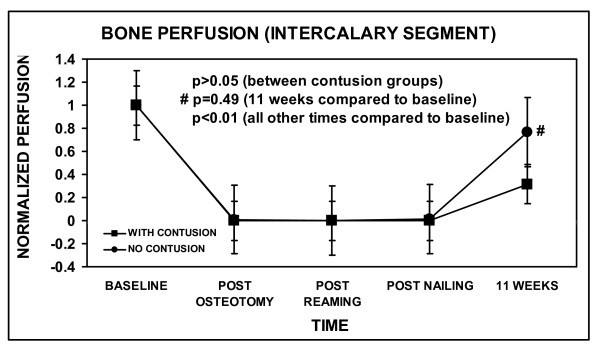
**Cortical bone blood flow of the intercalary segment of bone**. Values for each time point were the average reading from the three different laser doppler flowmetry measurement locations. The values at each time point were all normalized by dividing by the average value at baseline. The error bars indicate one standard error of the mean. No statistically significant differences between Contusion and No-Contusion groups were found (p > 0.05). All procedures at each time compared to baseline showed statistical differences (p < 0.01), except for 11 weeks in the No-Contusion group (#, p = 0.49).

Regarding overall tibial blood flow, all procedures in both groups were statistically different than baseline, except for 11 weeks in the No-Contusion group (p = 0.374) (Figure 4). Moreover, there was a statistically significant decrease in the overall bone perfusion following osteotomy in both groups (Figure 4), which was mainly due to the zero value of the intercalary segment (Figure 5). However, bone perfusion decreased to a larger extent in the Contusion group following osteotomy compared to the No-Contusion group. The difference between the two groups remained significant throughout the entire procedure. With regard to bone perfusion in the intercalary segment, all procedures were statistically different than baseline, except for 11 weeks in the No-Contusion group (p = 0.49) (Figure 5).

### Week 11 Data

There were no wound infections. All tibiae were healed clinically and radiographically at the time of harvesting. Muscle perfusion overall and in the zone of injury was statistically the same in both groups at 11 weeks, the level not being statistically different than baseline (Figure 2 and 3). Overall bone perfusion was greater in the No-Contusion group at 11 weeks, by which time it had returned to baseline levels (Figure 4). Site-specific analysis showed that the intercalary bone segment showed no statistical difference between the Contusion and No-Contusion groups at 11 weeks, although the No-Contusion group had returned to baseline levels by week 11 (Figure 5).

### Post Hoc Power Analysis

The post hoc power analysis at the 11-week mark for the muscle perfusion (overall) yielded 24% and for bone perfusion (intercalary segment) showed 38%. A high-powered statistical design is normally considered to be 80% or higher. Consequently, the number of specimens was not adequate to detect all statistical differences present.

## Discussion

Treatment of tibial shaft fractures associated with significant soft tissue injury remains challenging. This study attempted to simulate a high-energy injury resulting in an unstable fracture pattern with significant soft tissue injury. Therefore, a segmental fracture with a standardized muscle contusion was used. Reaming was performed so that its effects could be evaluated within an injured soft tissue envelope. Limited reaming was used because of the well-known detrimental effects of standard reaming [4,11-23]. Laser doppler flowmetry was employed to measure bone and muscle perfusion. This technique allows instantaneous blood flow measurements in vivo without the sacrifice of the experimental subject [29-31].

There was a profound hyperemic response in muscle perfusion after muscle contusion (Figure 2 and 3). This was pronounced within the zone of injury, although muscle perfusion at proximal and distal sites was also elevated compared to baseline. By the end of the initial procedure, muscle perfusion returned to baseline levels in Contusion and No-Contusion groups.

In the No-Contusion group, muscle perfusion overall and in the zone of injury did not statistically increase with reaming compared to baseline (Figure 2 and 3). This is consistent with previous studies [24,25]. In the Contusion group, immediately following reaming there was a decrease in muscle perfusion overall and in the zone of injury compared to peak values, but not with respect to baseline (Figure 2 and 3). This could be because muscle perfusion was at its maximal level following contusion, and the natural trend with injury is for muscle perfusion to decrease with time. If perfusion within the zone of injury was maximal following contusion, even reaming could not elevate the perfusion any further.

Bone perfusion decreased to a larger extent in the Contusion group throughout the initial procedure (Figure 4). It is well-known that if the endosteal blood supply is disrupted, as it is in a segmental tibia fracture, the surrounding soft tissues are responsible for the remaining blood supply to the bone [18-23]. Although muscle perfusion was increased in the Contusion group, tibial blood flow was decreased compared to the No-Contusion group. The authors postulate that this could be due to the initiation of inflammation with a resulting diversion of more blood flow for muscle repair, rather than delivering more blood to the injured bone. Moreover, although canal diameters were not statistically different, the low statistical power of the study may not have allowed detection of any real differences present between the two groups. Thus, it may be that the 15% difference in average canal diameter contributed to this finding. In addition, with the muscle being significantly injured, the functional capability of the capillaries is unknown. Therefore, while blood flow is increased, the bone may not be receiving the benefits of increased muscle perfusion. At 11 weeks, the overall bone perfusion in the Contusion group remained significantly lower than the No-Contusion group. This may suggest that soft tissue injury either had a sustained affect on cortical perfusion or had no influence on bone healing. This would need to be addressed in future studies using functional bone healing measurements not done presently.

The mechanical stiffness and strength of the bone-nail repair construct following diaphyseal fracture with simultaneous muscle contusion were not presently considered. A similar prior study assessed the effect of limited versus standard reaming on the 4-point bending stiffness and strength of nails used to repair segmental tibial shaft fractures in a series of canines, but without muscle contusion [24]. The results showed statistically significant decreases in repair construct stiffness (limited reaming, 30%; standard reaming, 46%) and strength (limited reaming, 35%; standard reaming, 22%) compared to intact contralateral tibias. Similar relative decreases in mechanical characteristics might be expected compared to intact tibias for the present specimens, with or without muscle contusion, had biomechanical tests also been performed. In addition, a recent study by Melnyk et al. quantified the revascularization process for diaphyseal fractures with and without surrounding soft tissue injury in a rat model [32]. They report that partly destroyed bone-soft tissue interaction resulted in only temporary reduction of extraosseous blood supply, which might not affect fracture healing. They also tested the mechanical properties of the repair constructs with and without surrounding soft tissue injury at four weeks post-operatively and found no statistical difference in failure load or flexural rigidity. The present authors, thus, suggest that the extent of blood perfusion into surrounding muscle and bone around the fracture site may not have any significant long-term affect on fracture healing and, hence, the mechanical stability of the bone-nail repair construct.

There were limitations to this study. Firstly, the choice of contusing the anterior compartment was arbitrary and may not be representative of all clinical scenarios. Posterior compartment injury is common and may significantly affect perfusion to the tibia. The degree of soft tissue injury will vary in the clinical setting.

Secondly, a small series of 11 animals was used due to funding and sheltering limitations. As such, the post hoc power analysis showed the study was underpowered with values of 24% (overall perfusion) and 38% (intercalary segment). Moreover, although canal diameters between groups were statistically not different, the low statistical power suggests that confounding effects due to canal size may have occurred.

Thirdly, a post hoc, rather than an a priori, statistical power analysis was performed. It is theoretically preferable to perform an a priori power analysis for initial study design to determine how many specimens should be included in an investigation to avoid statistical type II error. However, it is often difficult to do so because of large interspecimen variability and the unpredictability of outcome measures among specimens. Even when it seems possible to perform such a computation confidently, a post hoc power analysis is still necessary to confirm the statistical power of the study using the actual, rather than the predicted, results of the study.

Fourthly, a static 400 N load lasting 20 seconds was applied over a known contact area in order to create a reproducible model of muscle contusion that could be applied in a standardized manner in a laboratory setting. Although 400 N did alter the perfusion profile of bone and muscle, it is difficult to assess how representative this is of most high energy open tibial shaft fractures. Thus, higher load levels applied dynamically for a shorter time period would have more realistically simulated an impact injury. For instance, previous studies showed that a transverse load of about 750 N is required to fracture the diaphyseal region of a dog femur using an impact load applied at 3 m/s [33], whereas about 5270 N is required to fracture the proximal portion [34]. If an impact injury to the muscle was simulated at present, this may have increased the initial amount of blood loss and subsequently altered the current contusion group blood perfusion results. However, standardizing simulated impact injuries may not always be feasible because it requires recreating the same load level, load application time, and contact area for each animal. Therefore, a standardized static load approach was used in this investigation. The comparative nature of the study may allow the present results to be generalized to higher and dynamic loads.

Fifthly, the authors hypothesize that during the surgical procedure, the small differences in muscle and bone perfusion may have been due to the manipulation and/or injury of muscle bellies and adjacent soft tissues. The effect of this confounding factor, however, would need to be determined more conclusively in future investigations.

Sixthly, No-Contusion and Contusion groups both eventually healed. Thus, the clinical significance of the differences found in blood perfusion into muscle and bone is unknown. However, conditions under which adequate blood flow to surrounding bone and soft tissue can be maintained during trauma surgery and under which significant blood loss can be minimized, may possibly eliminate hemorrhagic or hypovolemic shock, reduce the need for post-operative blood infusion, increase fracture healing rate, and shorten patient recovery time [35].

Seventhly, the current surgical model simulated a segmental fracture of the tibial shaft, which is the least common type. Of all tibial shaft fractures, about 54% are simple fractures that have a spiral or oblique pattern, about 28% are wedge fractures, and about 18% are comminuted or segmental [36]. However, a segmental fracture was used because it was the easiest to simulate consistently from specimen to specimen in a research setting. Moreover, the current study using a segmental fracture in the presence of muscle contusion could then be compared with prior studies by some of the authors who also used a segmental fracture, but without muscle contusion [24,25].

Finally, although beyond the scope of the current study, future investigators could consider assessing the effect of standardized muscle contusion on the changes incurred on two other parameters of interest. Specifically, radiographs could be assessed and biomechanical tests could be performed to determine the amount of bone healing (or callus formation) at the fracture site [14,15]. Moreover, an evaluation could be done to determine whether muscle histology has fully recovered or whether the contusion site has been replaced totally or partially by scar tissue.

## Conclusions

This study showed that muscle injury may have a sustained, deleterious effect on bone perfusion during intramedullary nailing of a tibial fracture. This study was able to take some initial steps in the creation of a model which can lead to further assessment of the effects of muscle contusion on fracture healing by future investigators.

## List of Abbreviations

p: statistical significance criterion; PO BID: take medication orally or by mouth twice daily; SC OD: take medication under the skin once daily;

## Competing interests

No authors received personal financial benefit as a result of the study. In addition, no relationships to persons or organizations exist that compromise the integrity of this study.

## Authors' contributions

HK, TH, AT, and EHS were involved in developing the initial concept and study design. HK, TH, and AT obtained all the necessary supplies, managed animal care, performed surgeries, recorded outcome measurements, and did statistical analysis. HK and EHS wrote the initial draft of the paper. RZ extensively edited the paper, added new sections to the manuscript, formatted the figures, performed power analysis, updated the references, re-analyzed some data, and managed the submission to the journal for publication. EHS provided overall supervision, infrastructure support, and research funding for the project. All authors approve of this final manuscript version.

## References

[B1] Bhandari M Guyatt GH Swiontkowski MF Schemitsch EH Treatment of open fractures of the shaft of the tibia J Bone Joint Surg Br 2001 83 1 62 68 10.1302/0301-620X.83B1.10986 11245540

[B2] Keating JF O'Brien P Blachut P Meek RN Broekhuyse HM Locked intramedullary nailing with and without reaming for open fractures of the tibial shaft. A prospective, randomized study J Bone Joint Surg Am 1997 79 3 334 341 9070520 10.2106/00004623-199703000-00003

[B3] Bong MR Kummer FJ Koval KJ Egol KA Intramedullary nailing of the lower extremity: biomechanics and biology J Am Acad Orthop Surg 2007 15 2 97 106 17277256 10.5435/00124635-200702000-00004

[B4] Pape HC Giannoudis P The biological and physiological effects of intramedullary reaming J Bone Joint Surg Br 2007 89 11 1421 1426 10.1302/0301-620X.89B11.19570 17998175

[B5] Virkus WW Wakim EP Elstrom JA, Virkus WW, Pankovich AM Methods of fixation Handbook of Fractures 2006 New York, USA: McGraw-Hill 9 19

[B6] Anglen J Sanders R Tibial shaft fractures Trauma: Core Knowledge in Orthopaedics 2008 Philadelphia, USA: Mosby Elsevier 326 343

[B7] Anglen JO Blue JM A comparison of reamed and unreamed nailing of the tibia J Trauma 1995 39 2 351 355 10.1097/00005373-199508000-00027 7674406

[B8] Court-Brown CM Will E Christie J McQueen MM Reamed or unreamed nailing for closed tibial fractures. A prospective study in Tscherne C1 fractures J Bone Joint Surg Br 1996 78 4 580 583 8682824

[B9] Fairbank AC Thomas D Cunningham B Curtis M Jinnah RH Stability of reamed and unreamed intramedullary tibial nails: a biomechanical study Injury 1995 26 7 483 485 10.1016/0020-1383(95)00056-F 7493789

[B10] Henly MB Intramedullary devices for tibial fracture stabilization Clin Orthop Rel Res 1989 240 87 96 2917447

[B11] Danakwardt-Lilliestrom G Reaming of the medullary cavity and its effect on diaphyseal bone Acta Orthop Scand Suppl 1969 128 1 153 4192328 10.3109/ort.1969.40.suppl-128.01

[B12] Danakwardt-Lilliestrom G Lorenzi GL Olerud S Intramedullary nailing after reaming: an investigation on the healing process in osteotomized rabbit tibias Acta Orthop Scand Suppl 1970 134 1 78 5272157 10.3109/ort.1970.41.suppl-134.01

[B13] Danakwardt-Lilliestrom G Lorenzi GL Olerud S Intracortical circulation after intramedullary reaming with reduction of pressure in the medullary cavity: a microangiopathic study on the rabbit tibia J Bone Joint Surg Am 1970 52 7 1390 1394 5469193

[B14] Klein MPM Rhan BA Frigg R Kessler S Perren SM Reaming versus non-reaming in medullary nailing: interference with cortical circulation of the canine tibia Arch Orthop Trauma Surg 1990 109 6 314 316 10.1007/BF00636168 2073449

[B15] Schemitsch EH Kowalski MJ Swiontkowski MF Harrington RM Comparison of the effect of reamed and unreamed intramedullary nailing on blood flow in the callus and strength of union following fracture of the sheep tibia J Orthop Res 1995 13 3 382 389 10.1002/jor.1100130312 7602400

[B16] Schemitsch EH Kowalski MJ Swiontkowski MF Senft D Cortical bone blood flow in reamed and unreamed locked intramedullary nailing. A fractured tibia model in sheep J Orthop Trauma 1994 8 5 373 382 10.1097/00005131-199410000-00002 7996319

[B17] Sitter T Wilson J Browner B The effect of reamed versus unreamed nailing on intramedullary blood supply and cortical viability J Orthop Trauma 1990 4 2 232 10.1097/00005131-199004020-00074

[B18] Holden CEA The role of blood supply to soft tissue in the healing of diaphyseal fractures J Bone Joint Surg Am 1972 54 5 993 1000 5057112

[B19] Richards RR McKee MD Paitich CB Anderson GI Bertoia JT A comparison of the effects of skin coverage and muscle flap coverage on the early strength of union at the site of osteotomy after devascularization of a segment of canine tibia J Bone Joint Surg Am 1991 73 9 1323 1330 1918114

[B20] Richards RR Orsini EC Mahoney JL Verschuren R The influence of muscle flap coverage on the repair of devascularized tibial cortex: an experimental investigation in the dog Plast Reconstr Surg 1987 79 6 946 958 10.1097/00006534-198706000-00016 3588734

[B21] Richards RR Schemitsch EH Effect of muscle flap coverage on bone blood flow following devascularization of a segment of tibia: an experimental investigation in the dog J Orthop Res 1989 7 4 550 558 10.1002/jor.1100070413 2738772

[B22] Schemitsch EH Kowalski MJ Swiontkowski MF Soft tissue blood flow following reamed versus unreamed locked intramedullary nailing: A fractured sheep tibia model Ann Plastic Surg 1996 36 1 70 75 10.1097/00000637-199601000-00014 8722988

[B23] Triffitt PD Cieslak CA Gregg PJ A quantitative study of the routes of blood flow to the tibial diaphysis after osteotomy J Orthop Res 1993 11 1 49 57 10.1002/jor.1100110107 8423520

[B24] Hupel TM Aksenov SA Schemitsch EH Effect of limited and standard reaming on cortical bone blood flow and early strength of union following segmental fracture J Orthop Trauma 1998 12 6 400 406 10.1097/00005131-199808000-00006 9715447

[B25] Hupel TM Aksenov SA Schemitsch EH Muscle perfusion after intramedullary nailing of the canine tibia J Trauma 1998 45 2 256 262 10.1097/00005373-199808000-00009 9715181

[B26] Olson SA Open fractures of the tibial shaft J Bone Joint Surg Am 1996 78 9 1428 1437

[B27] Schneider E Michel MC Genge M Zuber K Ganz R Perren SM Loads acting in an intramedullary nail during fracture healing in the human femur J Biomech 2001 34 7 849 857 10.1016/S0021-9290(01)00037-9 11410169

[B28] Schneider E Michel MC Genge M Perren SM Bergmann G, Graichen F, Rohlmann A Loads acting on an intramedullary femoral nail Implantable Telemetry in Orthopaedics 1990 Berlin: Freie Universitat Berlin 221 227

[B29] Nilsson GE Tenland T Oberg PA A new instrument for continuous measurement of tissue blood flow by light beating spectroscopy IEEE Trans Biomed Eng 1980 27 1 12 19 10.1109/TBME.1980.326686 6444619

[B30] Nilsson GE Tenland T Oberg PA Evaluation of a laser Doppler flowmeter for measurement of tissue blood flow IEEE Trans Biomed Eng 1980 27 10 597 604 10.1109/TBME.1980.326582 6449469

[B31] Notzli HP Swiontkowski MF Thaxter ST Carpenter GK Wyatt R Laser Doppler flowmetry for bone blood flow measurements: helium-neon laser light attenuation and depth of perfusion assessment J Orthop Res 1989 7 3 413 424 10.1002/jor.1100070314 2703933

[B32] Melnyk M Henke T Claes L Augat P Revascularisation during fracture healing with soft tissue injury Arch Orthop Trauma Surg 2008 128 10 1159 1165 10.1007/s00402-007-0543-0 18094982

[B33] Benz G Höpfner H Göppl M Kallieris D Experimental studies of lateral stress to transverse fractured femora treated with external fixation Eur J Pediatr Surg 2006 16 5 343 347 10.1055/s-2006-924636 17160780

[B34] Hoshaw SJ Cody DD Saad AM Fyhrie DP Decrease in canine proximal femoral ultimate strength and stiffness due to fatigue damage J Biomech 1997 30 4 323 329 10.1016/S0021-9290(96)00159-5 9074999

[B35] Yach JD Sanders R Polytrauma considerations Trauma: Core Knowledge in Orthopaedics 2008 Philadelphia, PA USA: Mosby Elsevier 18 32

[B36] Appleton P Court-Brown CM Elstrom JA, Virkus WW, Pankovich AM Diaphyseal fracture of the tibia and fibula Handbook of Fractures 2006 New York, USA: McGraw-Hill 340 352

